# Use of crizotinib as neoadjuvant therapy for non‐small cell lung cancers patient with ROS1 rearrangement: A case report

**DOI:** 10.1111/1759-7714.14112

**Published:** 2021-08-17

**Authors:** Shikang Zhao, Shuai Zhu, Xi Lei, Dongbo Xu, Tao Shi, Qiusong Chen, Fan Ren, Gang Chen, Dingzhi Huang, Song Xu

**Affiliations:** ^1^ Department of Lung Cancer Surgery Tianjin Medical University General Hospital Tianjin China; ^2^ Tianjin Key Laboratory of Lung Cancer Metastasis and Tumor Microenvironment Lung Cancer Institute, Tianjin Medical University General Hospital Tianjin China; ^3^ Department of Pathology Tianjin Medical University General Hospital Tianjin China; ^4^ Precision Medicine Center Tianjin Medical University General Hospital Tianjin China; ^5^ Department of PET/CT Diagnostic Tianjin Medical University General Hospital Tianjin China; ^6^ Department of Thoracic Oncology Tianjin Medical University Cancer Institute and Hospital Tianjin China

**Keywords:** crizotinib, neoadjuvant, NSCLC, ROS1 rearrangements

## Abstract

Crizotinib showed significant antitumor effect in patients with advanced ROS1‐rearranged non‐small cell lung cancers (NSCLC). Most recently, many studies have explored the feasibility and efficacy of target therapy for perioperative application in NSCLC. Here, we describe a female patient who was diagnosed with stage IIIB lung adenocarcinoma exhibiting a CCDC6‐ROS1 rearrangement by high‐throughput sequencing. The tumor and lymph nodes showed durable response after the treatment of crizotinib. Given that a radiological downstaging was indicated, a right lower lobectomy and systemic lymphadenectomy were successfully performed. The pathological response was 60% and the tumor, nodes, and metastases (TNM) stage was ypT2bN0M0. The PD‐L1 expression and activity of immunological cells were also investigated.

## Graphical abstract

Comprehensive pathological evaluation. (a) Hematoxylin and eosin (HE) staining. Fibrosis and lymphocyte infiltration could be seen on the surgical specimen after crizotinib treatment. (b) Ki67 staining. (c) Multiple immunohistochemistry staining on CD163, CD8, PDL1, CD57, CD68, and PD1 before and after neoadjuvant crizotinib. (d) Quantitative analysis for staining data. *p < 0.05; **p < 0.01; ***p < 0.001, ns, not significant; Neo, neoadjuvant therapy.

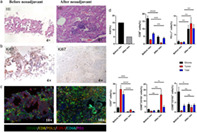

## INTRODUCTION

ROS proto‐oncogene 1 (ROS1) rearrangements occur in approximately 1%–2% of non‐small cell lung cancers (NSCLC), mainly existing in young, never, or light smokers with adenocarcinoma histology.^1^ Although patients with ROS1 rearrangements have similar clinical characteristics with anaplastic lymphoma kinase (ALK)‐positive NSCLC, the molecular mechanisms were different for ROS1 and ALK rearrangements. According to the impressive results of PROFILE 1001 study, crizotinib has been approved for treating metastatic NSCLCs patients with ROS1 rearrangement by the Food and Drug Administration (FDA) in 2015.^2^ In recent years, a series of studies and case reports have indicated the feasibility of the neoadjuvant targeted therapy for early stage resectable NSCLC patients with epidermal growth factor receptor (EGFR) and ALK mutations.^3^, ^4^, ^5^ However, there is no reports regarding crizotinib as neoadjuvant treatment for NSCLC patients with ROS1 rearrangement.

### CASE REPORT

**FIGURE 1:**
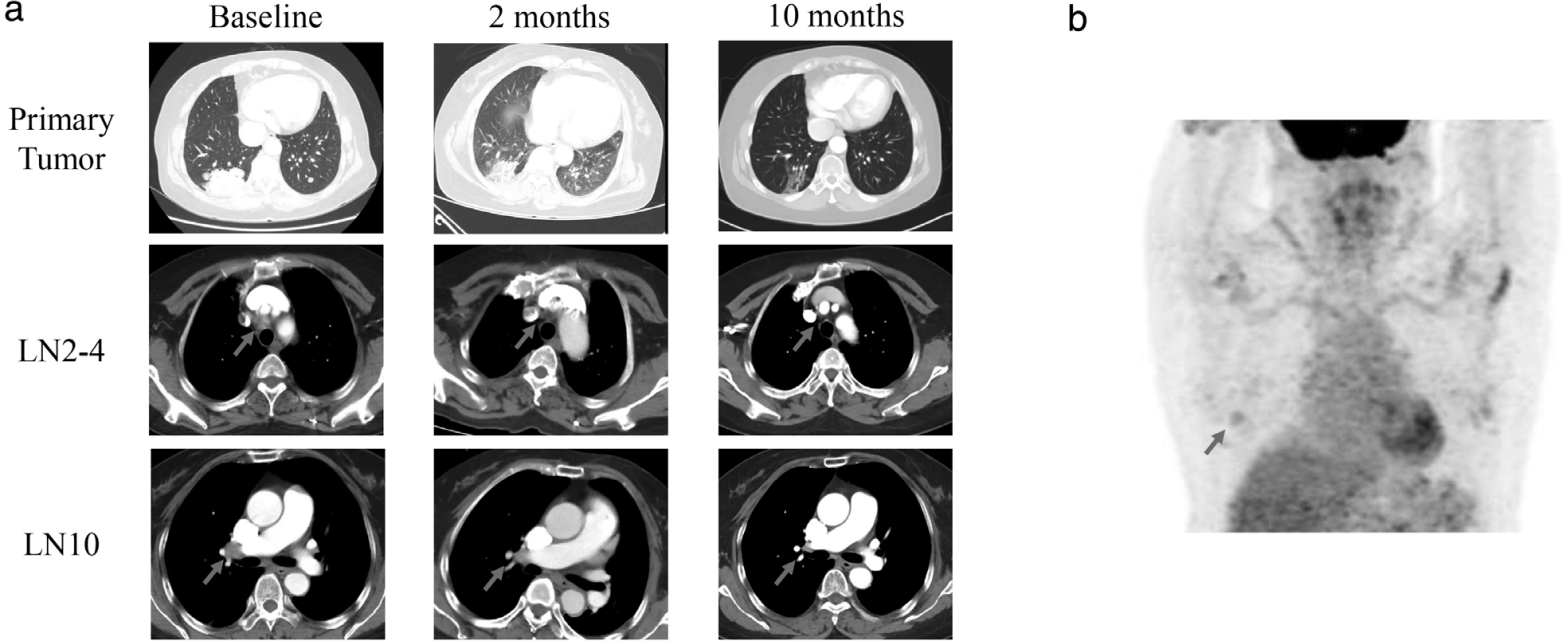
Images during neoadjuvant crizotinib treatment. (a) Enhanced chest CT images of right lung adenocarcinoma as well as lymph nodes (LN) prior, 2 and 8 months post crizotinib treatment; (b) PET‐CT image before the surgery

Here, we present the first case of neoadjuvant crizotinib in ROS1‐positive NSCLC patient. A 62‐year‐old female patient with no history of smoking was hospitalized because of 4 months of persistent back pain. She had a recent history of cerebral infarction. Enhanced computed tomography (CT) revealed a large mass with a diameter of 65 mm located in posterior basal segment of right lung, accompanied with enlarged right hilar, mediastinal lymph nodes, which were confirmed by positron emission tomography‐CT (PET‐CT) examination (cT3N2M0, IIIB, and AJCC 8th). The patient was given CT‐guided percutaneous biopsy for the lung mass. Pathological examination showed that the right lung lesion was invasive lung adenocarcinoma. We further performed a next‐generation sequencing with a 180‐gene panel (Genetron Health), and CCDC6‐ROS1 rearrangement was detected (Figure S1(a)). After a multiple disciplinary team (MDT) discussion, we thought this was a resectable stage IIIB case. Considering the recent cerebral infarction history at the time of admission and N2 LN involvement, we offered a treatment plan of chemoradiotherapy followed with surgical resection. However, the patient refused chemoradiotherapy and preferred target therapy. After obtaining informed consent from the patient, we prescribed crizotinib at a dosage of 250 mg twice per day. The lung tumor, as well as lymph nodes showed significant shrinkage after 2 months of treatment and exhibited durable response at the 10th month (Figure 1(a)). Ten months after the initial treatment, PET‐CT examination exhibited significantly decreased F^18^‐FDG uptake in right lesions with standard uptake value of 2.4 and no uptake was found in hilar and mediastinal lymph nodes (Figure 1(b)). A significant radiological response was achieved through neoadjuvant crizotinib with tumor shrinkage of almost 80%. The level of serum carcinoembryonic antigen (CEA) significantly decreased, however, still higher than the upper limit of normal (Figure S1(b)). During this course, the patient developed degree I dermatitis and moderate liver function injury related to the treatment. Crizotinib was discontinued for 2 weeks and restarted when liver function recovered after taking hepatoprotective drugs.

**FIGURE 2:**
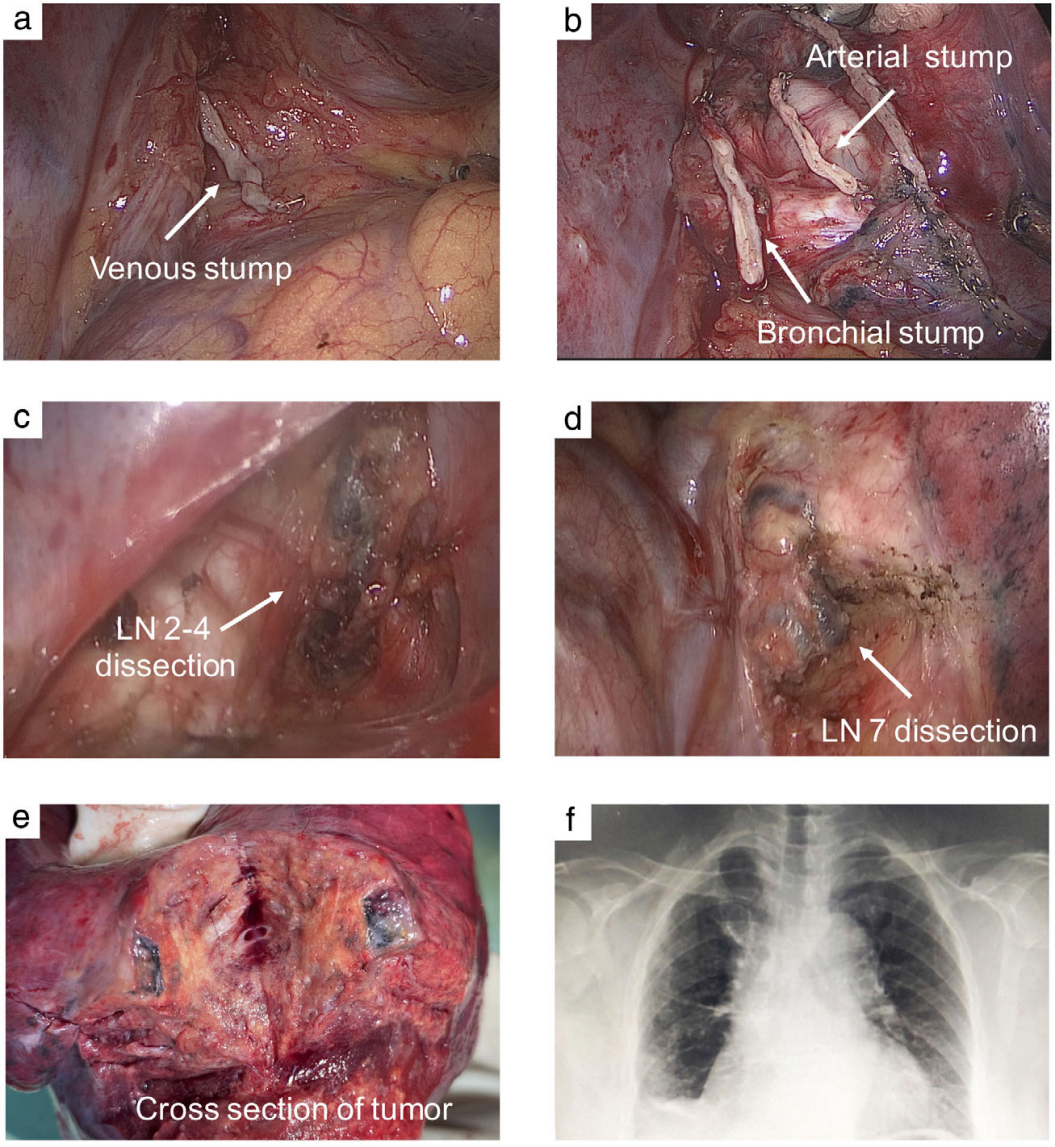
Intraoperative findings. (a),(b) Intraoperative arterial, venous and bronchial stumps; (c),(d) mediastinal lymph nodes; (e) cross section of the tumor; (f) chest radiograph after surgery

**FIGURE 3:**
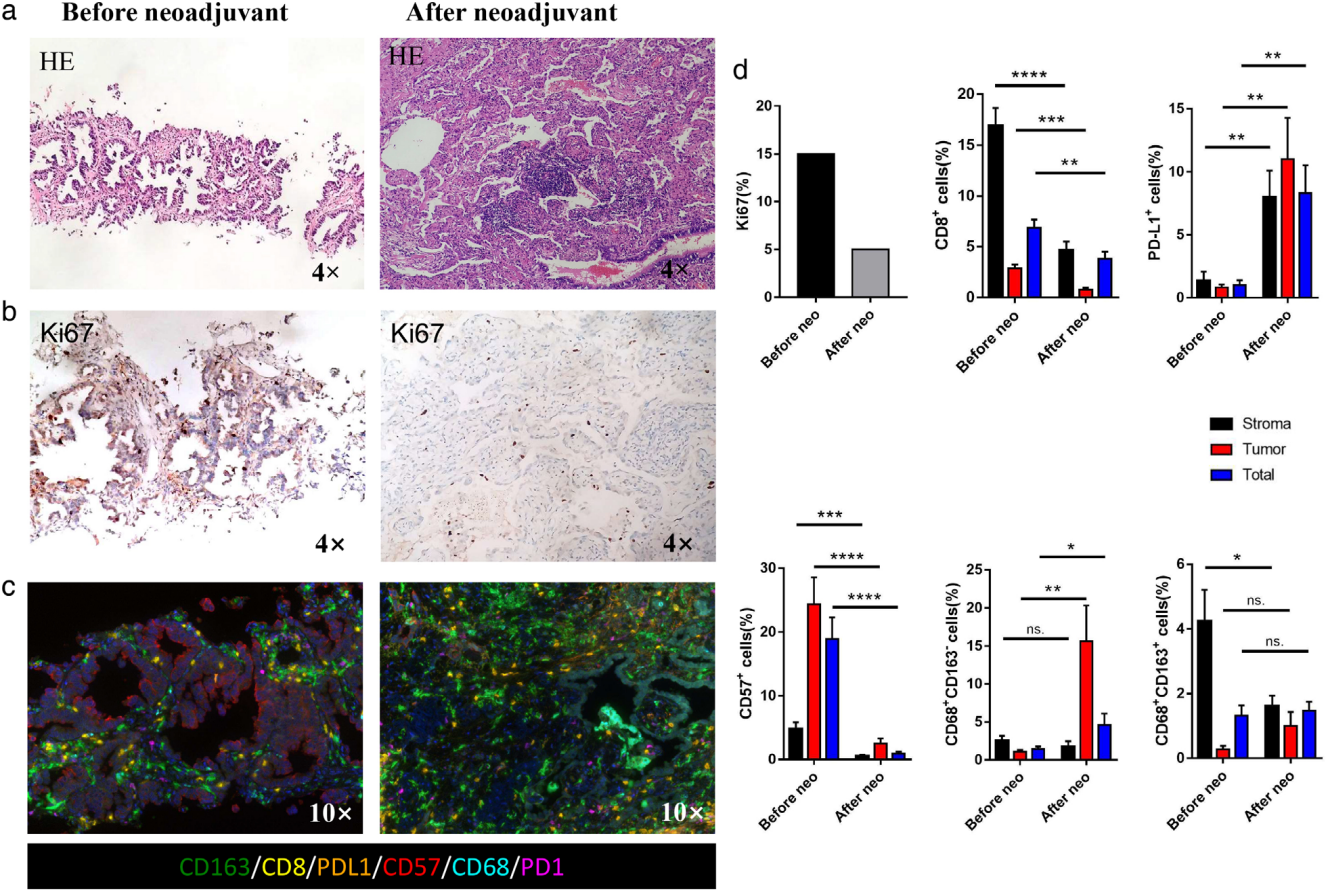
Comprehensive pathological evaluation. (a) Hematoxylin and eosin (HE) staining. Fibrosis and lymphocyte infiltration could be seen on the surgical specimen after crizotinib treatment. (b) Ki67 staining. (c) Multiple immunohistochemistry staining on CD163, CD8, PDL1, CD57, CD68, and PD1 before and after neoadjuvant crizotinib. (d) Quantitative analysis for staining data. *p < 0.05; **p < 0.01; ***p < 0.001. Neo, neoadjuvant therapy; ns, not significant

Given that the cerebral infarction had fully recovered and a radiological downstaging was indicated, right lower lobectomy and systemic lymphadenectomy were successfully performed 1 week after the last dose of crizotinib. The intraoperative findings with moderate adhesion were shown in Figure 2. Although the CT showed that the tumor was remarkably reduced, the postoperative pathological results showed that 40% of the tumor cells were still alive and were surrounded by prominent fibrosis, necrosis, inflammatory, and immune cells (Figure 3(a),(b)). After induction of crizotinib, the TNM stage was ypT2bN0M0 because of no metastasis for lymph nodes. Two months after surgery, the level of serum CEA returned to normal. Furthermore, we also performed a multiple immunohistochemistry (mIHC) staining (Genecast Biotechnology) on the biopsy tissue and surgical specimen to explore the alteration of PD‐L1 expression and activity of immunological cells. The results of mIHC suggested that PD‐L1 and CD68 were significantly increased, whereas CD8 and CD57 were decreased after neoadjuvant crizotinib treatment (Figure 3(c),(d)).

## DISCUSSION

To our knowledge, this is the first case of neoadjuvant crizotinib for NSCLC patients with ROS1 rearrangement. Previous clinical trials have already indicated the efficacy of neoadjuvant targeted therapy in the disease control and pathological downstaging in early stage EGFR and ALK‐positive NSCLC, and we also observed that crizotinib provided effective response in ROS1‐positive patients. More importantly, we found that the level of Ki67 expression is significantly decreased after the crizotinib‐targeted therapy, whereas 40% of the tumor cells in the postoperative tissues of the patients were still active, which implies the importance of radical resection after the induction of targeted therapy.

In addition, to further extend the value of our study, especially focusing on whether anti‐PD‐1/PD‐L1 immunotherapy is theoretically feasible in ROS1 gene mutation NSCLC patients, we performed the IHC staining of immune microenvironment before and after crizotinib therapy in this study. The staining demonstrated that PD‐L1 expression was dramatically increased, accompanied with M1 macrophage recruitment in the tumor after neoadjuvant treatment of crizotinib, while the treatment reduced the proportion of tumor infiltrated lymphocytes (TILs) and natural killer (NK) cells. In accordance with the previous reports, patients who acquired resistance to tyrosine kinase inhibitors (TKIs) express an increased level of PD‐L1 compared to TKI treatment naive status, which leads to a theoretical hypothesis of adoption of anti‐PD‐1 immunotherapy in the settings of TKI resistance. However, the evidence showed that NSCLC patients with acquired EGFR‐TKI resistance did not exhibit a robustly effective response against anti‐PD‐1 immunotherapy.^6^ The underlying mechanisms remain unclear. In our case, we observed that there was a significant reduction of TILs and NK cells, suggesting that immune microenvironment becomes more “cold” after crizotinib TKI treatment. Anti‐PD‐1 immunotherapy in combination with other treatments, which could recruit more TILs, is necessary for crizotinib TKI resistance patients.

Overall, further clinical trials with larger samples are needed to assess the effect of crizotinib for NSCLC patients with ROS1 rearrangement in the neoadjuvant setting as well as to explore immunotherapy‐based strategy in TKI‐resistant tumors.

## CONFLICT OF INTEREST

The authors declare no conflicts of interest.

## Supporting information


**Figure S1** Genetic mutation/tumor biomarker during neoadjuvant crizotinib treatment. (a) Sequencing read of CCDC6‐ROS1 rearrangement. (b) Dynamic alteration of CEA during crizotinib treatment.
