# Supplementary figures and images for: Use of crizotinib as neoadjuvant therapy for non‐small cell lung cancers patient with ROS1 rearrangement: A case report
Source: Thorac Cancer. 2021 Aug 17;12(20):2815–8. doi: 10.1111/1759-7714.14112 (PMC8520795; doi:10.1111/1759-7714.14112)

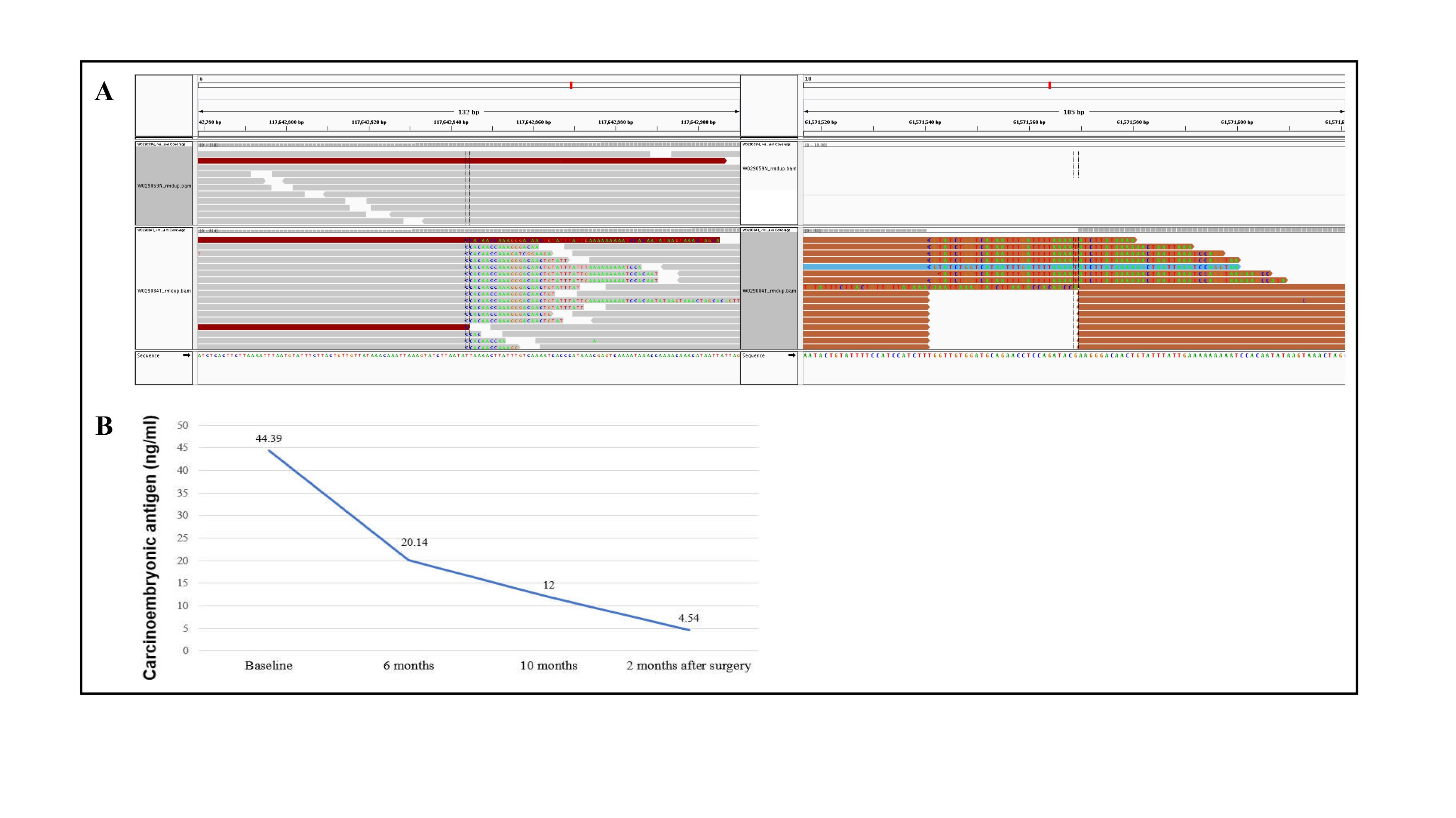

Supplement: Supplementary file 1 — Figure S1 Genetic mutation/tumor biomarker during neoadjuvant crizotinib treatment. (a) Sequencing read of CCDC6‐ROS1 rearrangement. (b) Dynamic alteration of CEA during crizotinib treatment. [file TCA-12-2815-s001.jpg]
